# Body image distress in women and men with advanced pancreatic cancer approaching death – post-hoc analyses from the MISTRAL-trial

**DOI:** 10.1007/s00520-026-11420-4

**Published:** 2026-10-27

**Authors:** Charlotte Goodrose-Flores, Stephanie E. Bonn, Linda Björkhem-Bergman, Kathrin Wode

**Affiliations:** 1 Division of Clinical Geriatrics, Department of Neurobiology, Care Sciences and Society, Karolinska Institutet, Huddinge, Sweden; 2 Division of Clinical Epidemiology, Department of Medicine, Karolinska Institutet, Solna, Sweden; 3 Palliative Medicine and R&D Unit, Stockholms Sjukhem, Mariebergsgatan 22, 112 19 Stockholm, Sweden; 4 Södersjukhuset, Stockholm, Sweden

**Keywords:** Cancer, Palliative, Body image

## Abstract

**Background:**

Weight loss is common in individuals with pancreatic cancer. The aim of the present study was to address how body image distress and weight worries change over time as death approaches in patients with pancreatic cancer, and whether there are gender differences.

**Methods:**

Post-hoc analyses were performed using data from the MISTRAL-trial. Body image distress and weight worries were assessed using the European Organisation for Research and Treatment of Cancer (EORTC) QLQ-PAN-26-questionnaire as part of the original trial. Data were used to assess body image distress and weight worries at time points corresponding to 1, 2, 3, 4, 5, and 6 months prior to death. Mixed-effects linear models with random intercepts were used to examine trajectories of body image distress and weight worries in women and men.

**Results:**

We analysed data from 125 participants, of whom 64 (51.2%) were women. Both body image distress and weight worries increased over time during the 6 months preceding death, β = 3.54, 95%CI: 1.67- 5.41; and β = 2.98, 95%CI: 0.53- 5.44, respectively. There were no statistically significant sex differences in the rate of change although women tended to report higher body image distress closer to death and men higher weight worries across all time points.

**Conclusion:**

Increased body image distress and weight loss concerns observed in both women and men with pancreatic cancer at the end of life highlight the importance of addressing these issues in palliative care.

## Introduction

Body image encompasses an individual’s perceptions, thoughts, and feelings about their own bodies [1]. It is shaped by the cultural context and cannot be meaningfully evaluated outside this framework. Hence, to understand body image issues and identify appropriate interventions, it is essential to consider the body image ideals of the respective culture [2, 3]. Women and men experience different body image concerns within the same cultural context. Previous research has shown that men are generally more satisfied with their bodies than women, even at higher Body Mass Index (BMI) levels [4]. In line with gender difference in the perception of body image, it is well established that women report smaller ideal female body silhouettes than men [5, 6].

Body image during cancer may arguably fall at the intersection between cultural body ideals and one’s pre-diagnosis body image, potentially complicating the understanding and management of body image during serious illness [7]. For example, women with breast cancer who reported a positive body image prior to cancer treatment dealt with the physical changes following the treatment much better than women who had reported body image distress [7]. Among individuals with rectal or colon cancer, women reported greater body image distress than men and those with colon cancer [8]. Interestingly, among individuals with advanced cancer of any type, men reported a more negative perception of weight loss compared to women [9]. How this discourse is translated into body image following weight loss in advanced cancer over time is underexplored and has received little attention.

Unintentional weight loss is common feature in cancer and a characteristic symptom of cancer cachexia [10]. In individuals with pancreatic cancer, 80% experience persistent weight loss [11]. Cancer related weight loss is not just a starvation-type malnutrition characterized by substrate and energy deficiency, but rather a complex metabolic condition involving abnormalities such as systemic inflammation [12]. Since maintaining body weight, regardless of weight pre-diagnosis, is crucial to extend months of living [13]. nutritional interventions are a key priority in cancer care [14]. For patients with pancreatic cancer, such interventions include pancreatic enzyme replacement therapy to reduce malabsorption, pharmacological treatments to alleviate nausea and vomiting, and dietary supplements to increase energy and nutritional intake [11, 15]. In addition, corticosteroids are often used as appetite stimulants. However, in a recent study, we were unable to demonstrate any beneficial effects of corticosteroid treatment on appetite or weight change during long-term follow-up in patients with pancreatic cancer [16].

In addition, corticosteroids are often used as appetite stimulants. However, in a recent study, we were unable to demonstrate any beneficial effects of corticosteroid treatment on appetite or weight change during long-term follow-up in patients with pancreatic cancer [16].

Considering the perception of weight loss and possibly differing body images between women and men with advanced cancer is likely an important perspective, when optimizing nutritional counselling in palliative care.

The aim of this study was to explore how body image distress, weight worries, and BMI change over time as patients with pancreatic cancer approach the end of life, and to examine potential differences between women and men.

## Methods

We have performed post-hoc analyses using data from the MISTRAL-trial, EU Clinical Trial Register, EudraCT Number 2014–004552-64, and registration NCT02948309, on October 28th, 2016, a Swedish multicentred, parallel group, double-blind, randomised, placebo-controlled clinical trial. The trial has been described in detail elsewhere [17]. In brief, MISTRAL was a multicentred, parallel group, double-blind, randomised, placebo-controlled clinical trial to which 290 patients with advanced pancreatic cancer were recruited. Participants were randomized 1:1 to either mistletoe extract or placebo in addition to comprehensive oncological treatment and palliative care during June 2016 to December 2021. Study assessments were performed at the time of randomization (baseline), after five to six weeks, and at two, three, four, six and nine months. On each occasion, participants measured their weight at the clinic and responded to the European Organisation for Research and Treatment of Cancer (EORTC) QLQ-PAN-26 questionnaire [18]. Since the MISTRAL-trial found no differences in either overall survival OS [19] nor quality of life [20]. between the treatment arms, mistletoe extract was not considered a confounder in the present study. Body weight (kg) was self-measured at home every week and recorded it in a study-specific patient diary. Information on age, height and sex at baseline was derived from the trial´s Case Report Forms The survival status of all randomized patients was checked before the end of the trial in January 2023. In the present study, we have analysed body image distress, weight worries, and BMI during the six months prior to death.

### Study population

**Fig. 1:**
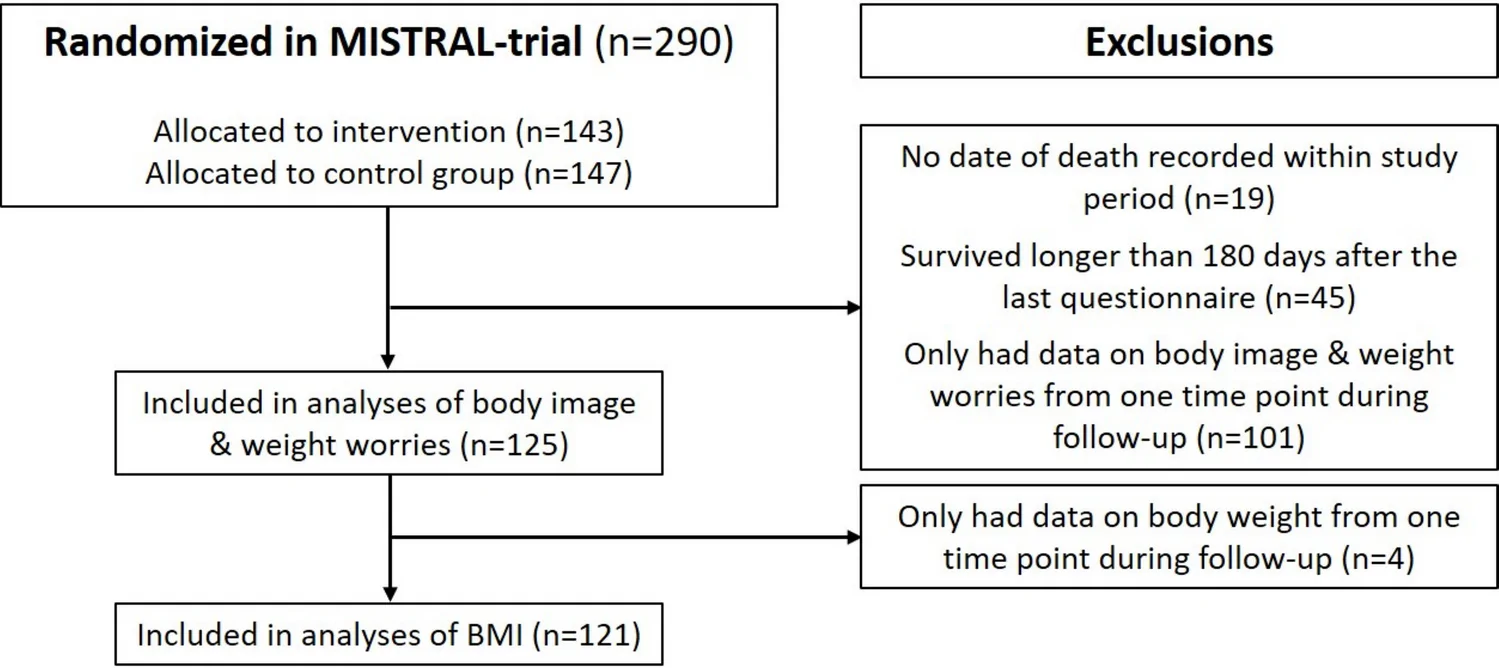
Flow-chart of exclusions from the MISTRAL population forming the present study´s study population

The study population included participants from the MISTRAL-trial that had died during the study period or within 180 days after study-end, and who had at least two assessments of body image and weight worries. From hereon we will refer to this as the “study population”. The exclusions are outlined in Fig. 1. The original MISTRAL-trial comprised 290 participants of which 19 did not have a date of death recorded within the study and were excluded. Participants with fewer than two assessments of body image six months before death were also excluded (*n* = 146). This group included the 45 participants having survived more than 180 days after the final study questionnaire and therefore lacking any assessment of body image during the six months before death. In analyses of weight change, participants with only one or zero recorded weight at the specified time points were further excluded (*n* = 4).

The use of corticosteroid as appetite stimulant in this cohort is described previously [16]. Five study participants reported using cannabinoids without a physician’s prescription. The indications for use, dosage, and duration of use are unknown (unpublished data). The prescription of cannabis-based medicines for symptom management remains uncommon in Swedish healthcare.

### Body image distress and weight worries

Body image distress and weight worries were addressed as by three questions included in the EORTC QLQ-PAN-26 questionnaire: “Have you felt physically less attractive as a result of your disease and treatment?” (item 48 in the questionnaire, body image), “Have you been dissatisfied with your body?” (item 49, body image), and “Have you worried about your weight being too low?” (item 41, weight worries). Participants responded to the questions using a 4-point Likert scale with response alternatives 1 ("Not at all"), 2 ("A little"), 3 ("Quite a bit"), and 4 ("Very much"). Variables were thereafter scored in accordance with the EORTC scoring manual [18]. Items 48 and 49 were included in a multi-item symptom scale of body image distress and raw scores were calculated as the average of the non-missing item responses and subsequently transformed to the 0–100 scale. In line with the scoring manual, the multi-item score was only calculated if at least half of the constituent items were completed, i.e. if participant had responded to at least one of the items. The single-item score of worries around weight (item 41) were linearly transformed to a standardized 0–100 scale, with a higher score indicating greater symptom burden. Higher scores indicate worse symptoms.

To address change in body image distress and weight worries as end-of-life approaches, we created variables of this at 30, 60, 90, 120, 150, and 180 days prior to death. Variables defining body image distress and weight worries, respectively, were created using the date of death and dates of responding to study questionnaires. Responses from questionnaires that fell on a date within two weeks from each time point (i.e. 30, 60, 90, 120, 150, and 180 days prior to the death date) were considered assessment of body image and weight worries at each certain time point. We will from here on denote these time points as one, two, three, four, five, and six months prior to death.

### Body weight

BMI at baseline was calculated using the weight and height measured at study inclusion. We also created variables defining body weight at one, two, three, four, five, and six months prior to death using weights from diaries recorded within 14 days of each time point. If multiple weight fell within the time frame, the weight recorded closest to the index date was use. If the participant did not have a recorded weight within this time frame, we used measured weights from study visits given that the measurement fell within the limit of 14 days. From this, BMI (kg/m^2^) at each month one to six was calculated using height measured at study inclusion.

### Statistical analyses

Characteristics of study participants are presented by mean values with and standard deviations (SD) for continuous variables with a normal distribution, median and interquartile range (IQR) for non-normally distributed variables, and number and percentages for categorical variables.

We used mixed-effects linear models with random intercepts to examine sex-differences in trajectories of body image distress, weight worries, and BMI during the six months prior to death. Time was modelled as a continuous variable (months before death), and the models included fixed effects for gender, time, and their interaction (gender × time) to assess whether changes over time differed by gender. The models included a random intercept for each individual to account for repeated measurements, allowing each person to have their own baseline level of the outcome (i.e., their estimated value at six months before death). Models were adjusted for age at study inclusion. All analyses were performed using STATA 17.0. The level of statistical significance was set at *p* < 0.05.

## Results

Characteristics of the study population are presented in Table 1. Among all 125 participants, 64 (51.2%) were women. Mean age was 68.4 (SD 10.1) years, mean BMI 23.5 (SD 3.7) kg/m^2^, and median time from diagnosis to randomization in the MISTRAL-trial was 37 days. There were no statistically significant differences in these variables between women and men. Median survival time from inclusion in the MISTRAL-trial was 6 (IQR: 4–8) months for this study population. Men had a statistically significantly shorter survival time than women, 5 (IQR: 3–8) vs. 7 (IQR: 5–9) (*p* = 0.03). Men where not eligible for palliative chemotherapy to a larger extent than women and received only best supportive at the time of inclusion (29.5% vs. 14.1%, respectively) (*p* = 0.04).

**Table 1:** Baseline characteristics of the study population by sex

	All (*n* = 125)		Men (*n* = 61)		Women (*n* = 64)		*P*^*1*^
	Mean	(SD)	Mean	(SD)	Mean	(SD)	
Age, years	68.4	(10.1)	69.3	(10.9)	67.5	(9.3)	0.31
BMI^2^, kg/m^2^	23.5	(3.7)	23.9	(3.6)	23.2	(3.7)	0.27
	Median	(IQR)	Median	(IQR)	Median	(IQR)	
Self-reported weight loss prior to diagnosis, kg	5	(2–10)	5	(3–10)	5	(1.5–8.5)	0.31
Time from diagnosis to randomization, days	37	(27–56)	38	(28–57)	36	(25–54)	0.39
Survival time from inclusion in MISTRAL, months	6	(4–8)	5	(3–8)	7	(5–9)	0.03
	n	(%)	n	(%)	n	(%)	
Received standard treatment							0.04
Palliative chemotherapy	98	(78.4)	43	(70.5)	55	(85.9)	
Best supportive care	27	(21.6)	18	(29.5)	9	(14.1)	

^1^Independent group t-test for normally distributed continuous variables, Wilcoxon rank-sum test for non-normally distributed continuous variables, and Chi-2 test for categorical variables comparing means and distribution, respectively, between sexes, ^2^Missing data *n* = 2, BMI at first study visit

**Fig. 2:**
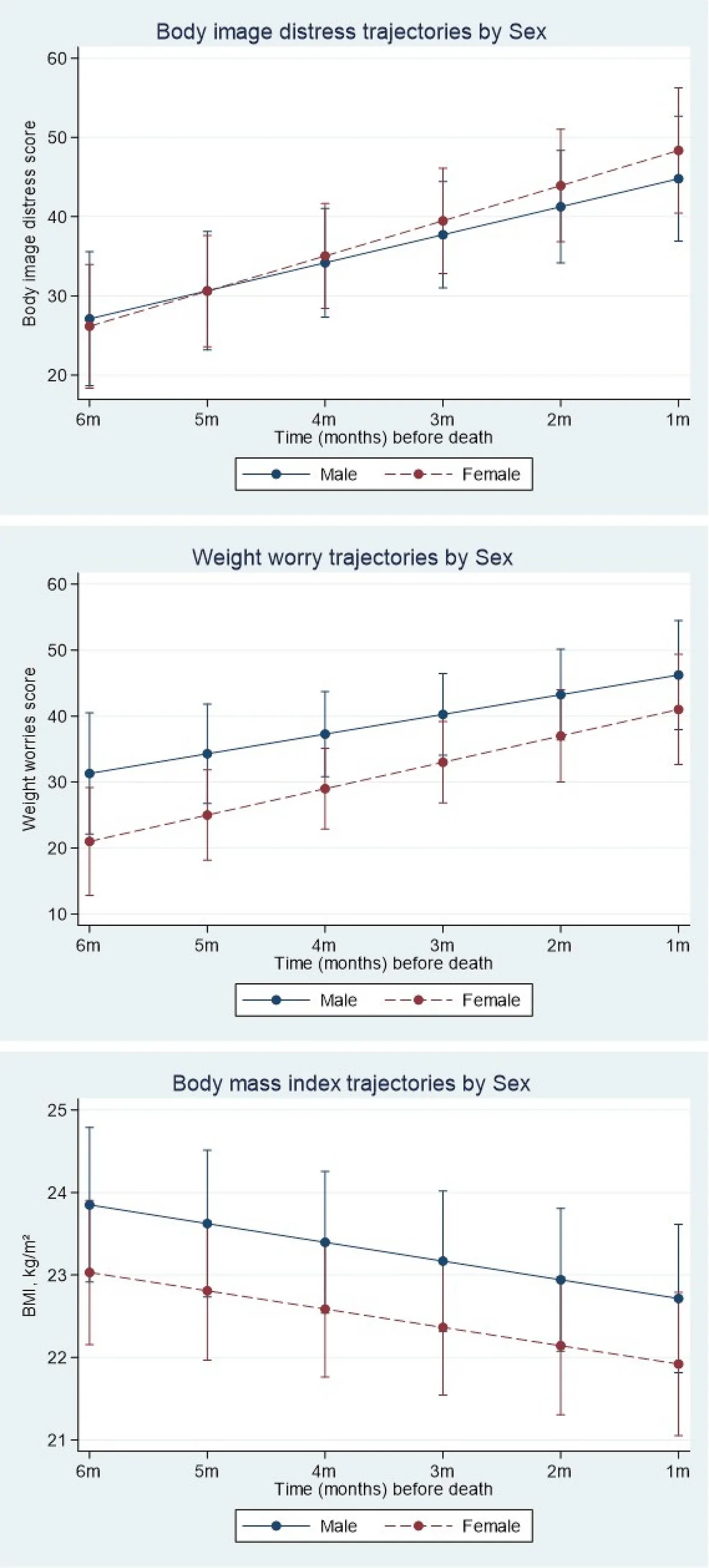
Graphical presentation of the results from linear mixed models with random intercept, adjusted for age. Predicted means at each time point is shown for women (dashed line) and men (solid line) separately for outcomes of weight worries, body image distress, and BMI over the last six months of life. Higher scores of body image distress and weight worries indicate worse symptoms

Table 2 presents the main effects from linear mixed models of weight worries, body image distress, and BMI over the last six months of life, and Table 3 shows the predicted means at each time point from the corresponding model. Figure 2 gives a graphical presentation of the modelled results.

**Table 2:** Results from mixed-effects linear regression models predicting body image, weight worries and BMI over the last six months of life, adjusting for age and allowing individual-level random intercepts (*n* = 349 observations from 125 individuals). Higher scores of body image and weight worries indicate worse symptoms

Predictor	β-coefficient	(95% CI)	*p*-value
Body Image distress score			
Female (vs. Male) at 6 months before death	−1.85	(− 15.00 to 11.30)	0.783
Change over time (per month before death)	**3.54**	**(1.67 to 5.41)**	**0.000**
Slope (Female#Month), rate of change over time	0.90	(− 1.64 to 3.45)	0.487
Change per year of age (continuous)	−0.31	(− 0.78 to 0.16)	0.190
Weight worries score			
Female (vs. Male) at 6 months before death	−11.33	(− 26.16 to 3.51)	0.134
Change over time (per month before death)	**2.98**	**(0.53 to 5.44)**	**0.017**
Slope (Female#Month), rate of change over time	1.02	(− 2.32 to 4.35)	0.551
Change per year of age (continuous)	0.24	(− 0.20 to 0.67)	0.288
Body mass index, kg/m^2^			
Female (vs. Male) at 6 months before death	−0.82	(− 2.19 to 0.54)	0.236
Change over time (per month before death)	**−0.23**	**(− 0.36 to − 0.09)**	**0.001**
Slope (Female#Month), rate of change over time	0.005	(− 0.18 to 0.19)	0.957
Change per year of age (continuous)	−0.0009	(− 0.06 to 0.06)	0.977

**Table 3:** Predicted means of body image distress, weight worries, and BMI by gender at each month during the six months prior to death. Values represent estimated marginal means with 95% confidence intervals from mixed-effects linear models adjusted for age. Higher scores of body image distress and weight worries indicate worse symptoms

	Predicted outcomes					
	Men		Women			
	Mean	(95% CI)	Mean	(95% SD)	Diff	(95% CI)
Body image distress score						
Month 6	27.10	(18.63 to 35.57)	26.16	(18.35 to 33.97)	−0.94	(− 12.46 to 10.57)
Month 5	30.64	(23.17 to 38.12)	30.60	(23.57 to 37.63)	−0.04	(− 10.31 to 10.22)
Month 4	34.18	(27.32 to 41.04)	35.04	(28.42 to 41.66)	0.86	(− 8.68 to 10.40)
Month 3	37.72	(31.00 to 44.44)	39.48	(32.83 to 46.12)	1.76	(− 7.70 to 11.23)
Month 2	41.26	(34.17 to 48.35)	43.92	(36.81 to 51.02)	2.66	(− 7.39 to 12.72)
Month 1	44.79	(36.90 to 52.69)	48.36	(40.43 to 56.28)	3.56	(− 7.64 to 14.77)
			Overall difference^1^:		1.36	(− 8.05 to 10.77)
Weight worries score						
Month 6	31.31	(22.14 to 40.48)	21.00	(12.83 to 29.17)	−10.31	(− 22.59 to 1.97)
Month 5	34.29	(26.76 to 41.83)	25.00	(18.15 to 31.85)	−9.29	(− 19.48 to 0.89)
Month 4	37.28	(30.83 to 43.72)	29.00	(22.89 to 35.11)	−8.28	(− 17.17 to 0.61)
Month 3	40.26	(34.06 to 46.46)	33.00	(26.84 to 39.16)	−7.26	(− 16.01 to 1.49)
Month 2	43.25	(36.34 to 50.13)	37.00	(30.02 to 43.99)	−6.24	(− 16.07 to 3.58)
Month 1	46.23	(37.96 to 54.50)	41.00	(32.64 to 49.36)	−5.23	(− 17.00 to 6.55)
			Overall difference^1^:		−7.71	(− 16.36 to 0.95)
Body mass index, kg/m^2^						
Month 6	23.85	(22.91 to 24.79)	23.03	(22.16 to 23.91)	−0.82	(− 2.10 to 0.46)
Month 5	23.62	(22.74 to 24.51)	22.81	(21.97 to 23.65)	−0.81	(− 2.04 to 0.41)
Month 4	23.40	(22.54 to 24.25)	22.59	(21.76 to 23.41)	−0.81	(− 2.00 to 0.38)
Month 3	23.17	(22.32 to 24.02)	22.36	(21.54 to 23.19)	−0.80	(− 1.99 to 0.38)
Month 2	22.94	(22.08 to 23.81)	22.14	(21.30 to 22.98)	−0.80	(− 2.01 to 0.41)
Month 1	22.71	(21.82 to 23.61)	21.92	(21.05 to 22.79)	−0.79	(− 2.05 to 0.46)
			Overall difference^1^:		−0.81	(− 1.99 to 0.38)

^1^Average difference between women and men over the whole time period

Body image distress scores showed a steady increase over time by 3.54 (95% CI: 1.67 to 5.41) points, in both sexes. There was no statistically significant difference between sexes in rate of increase (slope: 0.90 (95% CI: − 0.78 to 0.16), but the difference between predicted means is indicative of women experiencing worse symptoms closer to death compared to men.

Both women and men show steady increases in weight worry scores, indicating worse symptoms, when approaching death, with a statistically significant increase per month of 2.98 (95% CI: 0.53 to 5.44) points. There was no statistically significant difference in trajectory between the sexes (slope: 1.02 (95% CI: − 2.32 to 4.35). Men had higher predicted scores for weight worries at all time points, but the difference between the sexes was not statistically significant. However, the difference in predicted scores between women and men seem to decrease over time and become smaller when approaching death.

BMI is steadily decreasing in both sexes when approaching death, with an average monthly decrease of 0.23 (95% CI: − 0.36 to − 0.09) units of kg/m^2^. No difference in rate of decrease was seen between women and men (slope: 0.005 (95% CI: − 0.18 to 0.19). Although women have a lower predicted BMI than men at each time point, the differences were not statistically significant.

## Discussion

Both women and men with pancreatic cancer experienced a statistically significant increase in body image distress and concerns about weight loss during the disease trajectory toward the end of life. These experiences occurred in parallel with a decline in BMI.

Our findings suggest that body image distress and weight worries are relevant concerns among all patients with pancreatic cancer. While the trajectories of change in body image distress between women and men were not statistically significant in our data, likely due to the small sample size, it cannot be ruled out that it might be perceived differently between the genders. Women and men initially report similar levels of body image distress, but towards the end of life, women seem to experience a greater increase in distress than men. Individuals with pancreatic cancer continue to exist within a cultural context that places greater pressure on women than on men to maintain a thin body to be perceived attractive [21, 22].

We have previously observed a gender difference among patients with advanced cancer who had experienced weight loss [9]. Men reported a significantly increased body image distress following weight loss, whereas this was not observed in women. However, that study comprised patients with all types of cancer disease. In contrast to our previous findings, our present results showed that body image distress and weight worries increased in both women and men as they approached death. One possible explanation is that progression of pancreatic cancer is associated with a more rapid weight loss than most other cancer types and might be perceived as a more pronounced sign that death is approaching that outweighs the cultural ideals of female thinness.

Temporary and permanent body alterations, as well as changes in physical function, are common consequences of cancer, resulting from treatment, the disease itself, or both. This perspective on body image in cancer has been explored in different age groups and in relation to cancer types other than pancreatic, including breast, colorectal, and head and neck cancer. [23–28]. Age does not seem to be a determining factor in experiencing body image distress following a cancer diagnosis. Both adolescents and young adults with cancer [23] and older breast cancer survivors [24] have been shown to experience body image distress. Stomas, often a complication following colorectal cancer, are well-known for leading to body image distress [25] and it is common that treatments following head and neck cancer alter both appearance and functionality, which, in turn, affect body image [26] Bahrami et al. [28] found that regardless of cancer type (breast cancer, digestive cancer and blood cancer) most study participants experienced body image distress. Possible differences in body image between women and men have not been explored in any of these studies. Hegemonic masculinity, which is linked to physical strength and body image, has been explored in men with prostate cancer, and been found to have an important role as a coping mechanism post-treatment [27].

Our findings draw attention to an underexplored aspect of living with pancreatic cancer in the palliative phase. Our results highlight that physical distress and decline, reflected in body image distress, weight worries, and decreasing BMI, are concerns that extend beyond the physical effects of treatments such as chemotherapy, surgery, radiation, or other treatment modalities. This underpins the importance of healthcare professionals recognizing and addressing such concerns in clinical practice, as they may significantly impact quality of life.

This study has several strengths and limitations. Firstly, one strength of the study is that body image distress and weight worries were assessed using a well-established questionnaire, and that this is the first time that body image distress has been studied over time in individuals with advanced pancreatic cancer. The questionnaire, EORTC PAN-26, was first psychometrically validated in 2017, [18, 29] but has been applied in clinical research for a considerable period of time [29, 30].

Secondly, the data is based on a randomized controlled trial, with a thorough follow-up of all participants which enabled us to address body image distress and weight worries during the end of life although this was not the primary aim of the trial. However, we performed a post-hoc analysis, which was not pre-specified and should therefore be considered exploratory and interpreted with caution. The post-hoc design also increases the risk of our study being underpowered, i.e. limiting the statistical power to detect differences. Another limitation is the potential for confounding, as factors not accounted for in the analyses may have influenced the observed data. Our findings should therefore be interpreted with caution.

A suggested, more suitable design, for a future study, would be a prospective longitudinal cohort of individuals with advanced cancer. However, it is important from an ethical point of view to use and analyze data that has been collected. While the results do not directly benefit the participants, the results may have the potential to improve care for future individuals in similar medical situations [31].

A third strength is that study participants were recruited from across Sweden, enhancing the representativeness of the study population and supporting the extrapolation of results to a broader group of patients with pancreatic cancer in the palliative phase. However, the patients included in the MISTRAL-trial may represent a selected group, as they felt sufficiently robust to consider participating in a study despite being in a palliative phase. Nevertheless, the selection of individuals to the present study included only patients with a short remaining life span. Moreover, the study participants may not be representative of patients with other types of cancer in palliative care, and the generalizability of the findings may therefore be limited.

In conclusion, the increased body image distress and weight loss concerns observed in both women and men with pancreatic cancer at the end of life highlight the importance of addressing these issues in palliative care. Future studies on body image and weight perception in individuals with advanced cancer should also consider potential gender differences.

## Data Availability

The datasets used and/or analyzed during the current study are available from the corresponding author on reasonable request.

## Declarations

### Trial registration of the MISTRAL-trial

EudraCT 2014–004552–64, NCT02948309.
